# A new quadrannulate species of *Orobdella* (Hirudinida, Arhynchobdellida, Orobdellidae) from western Honshu, Japan

**DOI:** 10.3897/zookeys.553.6723

**Published:** 2016-01-14

**Authors:** Takafumi Nakano

**Affiliations:** 1 Department of Science Education, Graduate School of Education, Hiroshima University, Higashihiroshima 739-8524, Japan; 2 Department of Zoology, Graduate School of Science, Kyoto University, Kyoto 606-8502, Japan

**Keywords:** Hirudinea, Orobdella, new species, gastroporous, molecular phylogeny, Japan

## Abstract

A new quadrannulate species of *Orobdella* Oka, 1895, *Orobdella naraharaetmagarum* **sp. n.**, from the mountainous region of western Honshu, Japan is described. *Orobdella naraharaetmagarum* is a small species with a body length of less than 5 cm. Phylogenetic analyses using nuclear 18S rRNA and histone H3, as well as mitochondrial cytochrome *c* oxidase subunit I, tRNA^Cys^, tRNA^Met^, 12S rRNA, tRNA^Val^, 16S rRNA, tRNA^Leu^ and NADH dehydrogenase subunit 1 markers indicated that the present new species is the sister species of the quadrannulate *Orobdella esulcata* Nakano, 2010. Furthermore, mitochondrial DNA genealogy within *Orobdella naraharaetmagarum* demonstrated that this new species is divided into eastern and western lineages.

## Introduction

The terrestrial macrophagous leech genus *Orobdella* Oka, 1895 contains 12 species that are distributed throughout the Japanese Archipelago, Korean Peninsula, and Taiwan (Nakano 2014, Nakano and Lai 2012, Nakano and Seo 2014). These 12 species are split into three groups based on their mid-body somite annulation: seven species in the quadrannulate (four annuli) group, four in the sexannulate (six annuli) group, and one octannulate (eight annuli) species.

*Orobdella* leeches had been considered large species, with body lengths reaching to 10 cm or greater (Sawyer 1986). In recent years, however, small mature leeches belonging to this genus have been discovered in Japan and described as new species: *Orobdella koikei* Nakano, 2012b from Hokkaido, and *Orobdella masaakikuroiwai* Nakano, 2014 from central Honshu. The bodies of mature individuals of these two species are shorter than 4 cm. Both species possess mid-body somites that are quadrannulate. Nakano (2014) suggested that differences in the body lengths of mature leeches might enable more than one species of *Orobdella* to coexist in the same region.

Additional small *Orobdella* leeches were collected from Chugoku District, western Honshu, Japan. The body lengths of the specimens were less than 5 cm. Nevertheless, a few individuals were regarded as mature leeches because they possessed an obvious clitellum. These specimens are described here as a new species. In addition, the phylogenetic position of this new species was estimated using nuclear 18S rRNA and histone H3, as well as mitochondrial cytochrome *c* oxidase subunit I, tRNA^Cys^, tRNA^Met^, 12S rRNA, tRNA^Val^, 16S rRNA, tRNA^Leu^, and NADH dehydrogenase subunit 1 sequence data.

## Materials and methods

### Sampling and morphological examination

**Figure 1.**
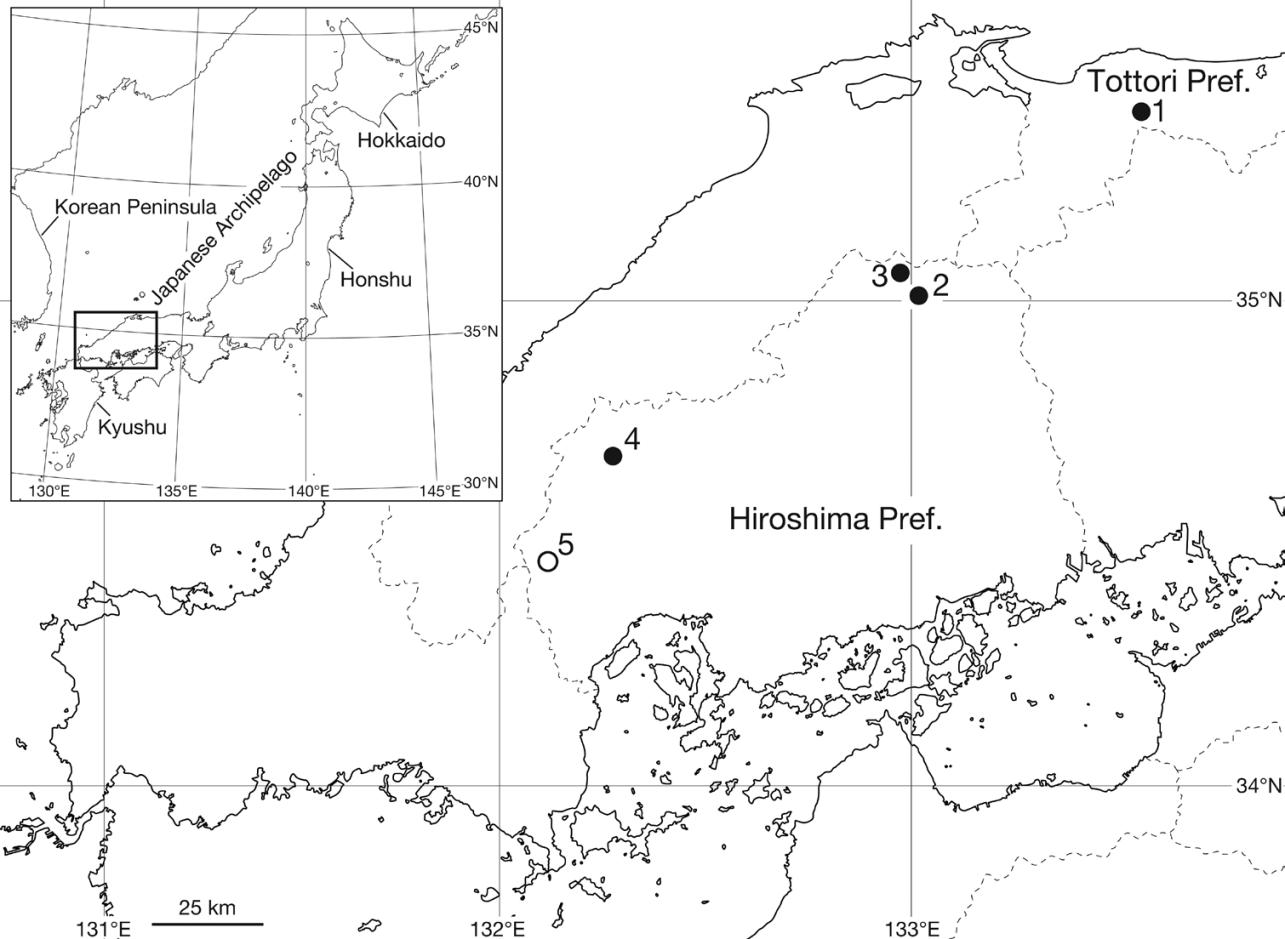
Map showing the collection localities of the specimens examined in this study. Open circle (**5**) indicates the type locality of the new species, *Orobdella naraharaetmagarum* sp. n., and closed circles (**1–4**) indicate additional localities.

Leeches were collected from five localities in Chugoku district, western Honshu, Japan (Fig. 1). When possible, elevation and geographical coordinates for localities were obtained using a Garmin eTrex® GPS unit.

Almost all of the specimens were relaxed by the gradual addition of absolute ethanol (EtOH) to freshwater. For DNA extraction, botryoidal tissue was removed from the posterior part of the body around the caudal sucker of every specimen, and then preserved in absolute EtOH. The remainder of the body was fixed in 10% formalin and preserved in 70% EtOH. Four measurements were taken: body length (BL) from the anterior margin of the oral sucker to the posterior margin of the caudal sucker, maximum body width (BW), caudal sucker length (CL) from the anterior to the posterior margin of the sucker and caudal sucker width (CW) from the right to the left margin of the sucker. Examination, dissection, and drawing of the specimens were conducted using a stereoscopic microscope with a drawing tube (Leica M125). Specimens used in this study have been deposited in the Zoological Collection of Kyoto University (KUZ).

The numbering convention is based on Moore (1927): body somites are denoted by Roman numerals, and the annuli in each somite are given alphanumeric designations.

### PCR and DNA sequencing

The extraction of genomic DNA from botryoidal tissues preserved in absolute EtOH followed Nakano (2012b). Primer sets for the PCR and cycle sequencing (CS) reactions used in this study were as follows: for 18S rRNA, A and L (PCR and CS), C and Y (PCR and CS), as well as O and B (PCR and CS) (Apakupakul et al. 1999); for histone H3 (H3), H3aF and H3bR (PCR and CS) (Colgan et al. 1998); for cytochrome *c* oxidase subunit I (COI), LCO 1490 and HCO 2198 (PCR and CS) (Folmer et al. 1994), and LCO-in (Nakano 2012b) and HCO-outout (Nakano 2012a) (PCR and CS), or LCO-inerpo2 (5'-GCTATTACAATATTACTTACAGATCG-3'; this study) and HCO-out (Nakano 2012b) (PCR and CS); for tRNA^Cys^, tRNA^Met^, 12S rRNA, tRNA^Val^ and 16S rRNA (tRNA^Cys^–16S), 12SA-out and 12SB-in (PCR and CS), and 12SA-in and 12SB-out (Nakano 2012b) or 12SB-outin (5'-AAAGGTACGAATATATTTAC-3'; this study) (PCR and CS); for tRNA^Leu^ and NADH dehydrogenase subunit 1 (ND1) (tRNA^Leu^–ND1), LDN3000 and HND1932 (PCR and CS) (Light and Siddall 1999). The PCR reactions and DNA sequencing were performed using the modified method mentioned in Nakano (2012a). The PCR reactions were performed using a GeneAmp PCR System 2700 and a GeneAmp PCR System 9700 (Applied Biosystems) as well as a T100 Thermal Cycler (Bio-Rad). The PCR mixtures were heated to 94 °C for 5 min, followed by 35 cycles at 94 °C (10 s each), 52 °C for 18S and H3, 60 °C, and 44 °C, respectively, for the anterior, and posterior parts of tRNA^Cys^–16S or 42 °C for COI and tRNA^Leu^–ND1 (20 s), and 72 °C (42 s each), and a final extension at 72 °C for 6 min. The sequencing mixtures were heated 96 °C for 2 min, followed by 40 cycles at 96 °C (10 s each), 50 °C (5 s each) and 60 °C (48 s each). The obtained sequences were edited using DNA BASER (Heracle Biosoft S.R.L.). The DNA sequences listed in Table 1 were newly obtained in this study and were deposited with the International Nucleotide Sequence Database Collaboration (INSDC) through the DNA Data Bank of Japan (DDBJ).

**Table 1.** Samples used for the phylogenetic analyses. The information on the vouchers is accompanied by the collection locality numbers for *Orobdella naraharaetmagarum* sp. n. (see Fig. 1) and the INSDC accession numbers. Sequences marked with an asterisk were obtained for the first time in the present study. Acronyms: KUZ, the Zoological Collection of Kyoto University; UNIMAS, the Universiti Malaysia Sarawak.

Species	Voucher (locality number)	18S	Histone H3	COI	tRNA^Cys^–16S	tRNA^Leu^–ND1
*Orobdella naraharaetmagarum* sp. n.	KUZ Z1360 (4)			LC087131*	LC087130*	LC087132*
*Orobdella naraharaetmagarum* sp. n.	KUZ Z1380 (2)			LC087134*	LC087133*	LC087135*
*Orobdella naraharaetmagarum* sp. n.	KUZ Z1535 (1)			LC087137*	LC087136*	LC087138*
*Orobdella naraharaetmagarum* sp. n.	KUZ Z1582 Paratype (5)			LC087140*	LC087139*	LC087141*
*Orobdella naraharaetmagarum* sp. n.	KUZ Z1652 Holotype (5)	LC087143*	LC087145*	LC087144*	LC087142*	LC087146*
*Orobdella naraharaetmagarum* sp. n.	KUZ Z1654 Paratype (5)			LC087148*	LC087147*	LC087149*
*Orobdella naraharaetmagarum* sp. n.	KUZ Z1655 (3)			LC087151*	LC087150*	LC087152*
*Orobdella dolichopharynx* Nakano, 2011b	KUZ Z120 Holotype	AB663665	AB698876	AB679680	AB679681	AB828558
*Orobdella esulcata* Nakano, 2010	KUZ Z29 Holotype	AB663655	AB698873	AB679664	AB679665	AB828555
*Orobdella ijimai* Oka, 1895	KUZ Z110 Topotype	AB663659	AB698877	AB679672	AB679673	AB828559
*Orobdella kawakatsuorum* Richardson, 1975	KUZ Z167 Topotype	AB663661	AB698878	AB679704	AB679705	AB828561
*Orobdella ketagalan* Nakano & Lai, 2012	KUZ Z208 Holotype	AB704785	AB704786	AB704787	AB828582	AB828563
*Orobdella koikei* Nakano, 2012b	KUZ Z156 Holotype	AB698883	AB698882	AB679688	AB679689	AB828560
*Orobdella masaakikuroiwai* Nakano, 2014	KUZ Z694 Holotype	AB938003	AB938013	AB938006	AB937997	AB938016
*Orobdella mononoke* Nakano, 2012a	KUZ Z224 Holotype	AB698868	AB698869	AB698866	AB698867	AB828564
*Orobdella octonaria* Oka, 1895	KUZ Z181 Topotype	AB698870	AB698871	AB679708	AB679709	AB828562
*Orobdella shimadae* Nakano, 2011b	KUZ Z128 Holotype	AB663663	AB698875	AB679676	AB679677	AB828557
*Orobdella tsushimensis* Nakano, 2011a	KUZ Z134 Holotype	AB663653	AB698872	AB679662	AB679663	AB828554
*Orobdella whitmani* Oka, 1895	KUZ Z45 Topotype	AB663657	AB698874	AB679668	AB679669	AB828556
*Erpobdella japonica* Pawłowski, 1962	KUZ Z178	AB663648	AB698879	AB679654	AB679655	AB828542
*Gastrostomobdella monticola* Moore, 1929	UNIMAS/A3/BH01/10	AB663649	AB698880	AB679656	AB679657	AB828543
*Mimobdella japonica* Blanchard, 1897	KUZ Z179	AB663650	AB698881	AB679658	AB679659	AB828544
*Odontobdella blanchardi* (Oka, 1910)	KUZ Z180	AB663651	AB938012	AB938004	AB937995	AB938014

### Molecular phylogenetic and genetic distance analyses

Eighty published sequences were obtained from the INSDC for use in molecular phylogenetic analyses (Table 1). In addition to 12 known *Orobdella* species, the following four erpobdelliform species were used as outgroup taxa: *Erpobdella japonica* Pawłowski, 1962 (Erpobdellidae), *Gastrostomobdella monticola* Moore, 1929 (Gastrostomobdellidae), *Mimobdella japonica* Blanchard, 1897, and *Odontobdella blanchardi* (Oka, 1910) (both Salifidae).

The phylogenetic position of the newly identified *Orobdella* species within the genus was estimated based on 18S, H3, COI, tRNA^Cys^–16S, and tRNA^Leu^–ND1 sequences. The alignments of H3 and COI were trivial, as no indels were observed. 18S, tRNA^Cys^–16S, and tRNA^Leu^–ND1 were aligned using MAFFT v. 7.245 L-INS-i (Katoh and Standley 2013). The lengths of the 18S, H3, COI, tRNA^Cys^–16S, and tRNA^Leu^–ND1 sequences were 1,844, 328, 1,267, 1,120, and 633 bp, respectively. The concatenated sequences yielded 5,192 bp of aligned positions.

Phylogenetic trees were constructed using maximum likelihood (ML) and Bayesian inference (BI). ML phylogenies were constructed using RAxML v. 8.1.5 (Stamatakis 2014) with the substitution model set as GTRCAT, immediately after nonparametric bootstrapping (Felsenstein 1985) conducted with 1,000 replicates. The best-fit partitioning scheme for the ML analyses was identified with the Akaike information criterion (Akaike 1974) using PartitionFinder v. 1.1.1 (Lanfear et al. 2012) with the “greedy” algorithm: 18S/the 1^st^ and 2^nd^ positions of H3/the 3^rd^ position of H3/the 1^st^ position of COI/the 2^nd^ position of COI/the 3^rd^ positions of COI and ND1/the 1^st^ position of ND1/the 2^nd^ position of ND2/12S/16S/tRNA^Cys^, tRNA^Met^, tRNA^Val^ and tRNA^Leu^.

BI and Bayesian posterior probabilities (BPPs) were estimated using MrBayes v. 3.2.5 (Ronquist et al. 2012). The best-fit partition scheme and models for each partition were selected based on the Bayesian information criterion (Schwarz 1978) using PartitionFinder with the “greedy” algorithm: for 18S and the 1^st^ position of H3, K80+I; for the 2^nd^ position of H3, JC69; for the 3^rd^ position of H3, HKY85; for the 1^st^ position of COI, GTR+G; for the 2^nd^ positions of COI and ND1, HKY85+I; for the 3^rd^ positions of COI and ND1 plus 16S, HKY85+I+G; and for the 1^st^ position of ND1, and 12S, tRNA^Cys^, tRNA^Met^, tRNA^Val^ and tRNA^Leu^, GTR+I+G. Two independent runs of four Markov chains were conducted for 12 million generations, and the tree was sampled every 100 generations. The parameter estimates and convergence were checked using Tracer v. 1.6.0 (Rambaut and Drummond 2009) and the first 30,001 trees were discarded based on these results.

The phylogenetic relationships within the available *Orobdella* materials were estimated based on sequences of COI, tRNA^Cys^–16S and tRNA^Leu^–ND1. tRNA^Cys^–16S and tRNA^Leu^–ND1 were aligned using MAFFT L-INS-i. The lengths of the COI, tRNA^Cys^–16S, and tRNA^Leu^–ND1 sequences were 1,267, 634, and 1,107 bp, respectively. The concatenated sequences yielded 3,008 bp of aligned positions. ML phylogenies were constructed in RAxML with the substitution model set as GTRCAT, immediately after nonparametric bootstrapping conducted with 1,000 replicates. The best-fit partitioning scheme was identified with the Akaike information criterion using PartitionFinder with the “greedy” algorithm: the 1^st^ position of COI/the 2^nd^ positions of COI and ND1/the 3^rd^ positions of COI and ND1/the 2^nd^ position of ND1/the 1^st^ position of ND1/tRNA^Cys^, tRNA^Met^, tRNA^Val^, tRNA^Leu^/12S/16S. BI and BPPs were estimated using MrBayes. The best-fit partition scheme and models for each partition were selected based on the Bayesian information criterion using PartitionFinder with the “greedy” algorithm: for the 1^st^ positions of COI and ND1, GTR+I+G; for the 2^nd^ positions of COI and ND1, F81+I; for the 3^rd^ positions of COI and ND1 plus 16S, HKY+G; tRNA^Cys^, tRNA^Met^, 12S, tRNA^Val^ and tRNA^Leu^, GTR+I+G. Two independent runs of four Markov chains were conducted for 10 million generations and the tree was sampled every 100 generations. The parameter estimates and convergence were checked using Traced, and the first 25,001 trees were discarded based on these results.

Nodes with bootstrap support (BS) values higher than 70% were considered sufficiently resolved (Hillis and Bull 1993). Nodes with BPPs higher than 95% were considered statistically significant (Leaché and Reeder 2002).

Pairwise comparisons of uncorrected *p*-distances for seven COI sequences (1,266 bp) obtained from specimens of the studied species and *Orobdella esulcata* Nakano, 2010 were calculated using MEGA6.06 (Tamura et al. 2013).

## Taxonomy

### Family Orobdellidae Nakano et al., 2012

http://zoobank.org/5F5BABE8-BD26-4FC7-9593-F73E62E26122

### Genus *Orobdella* Oka, 1895

http://zoobank.org/FA8333ED-8C17-41FD-AFC1-62A4F98D4AC1

#### Orobdella naraharaetmagarum sp. n.

Taxon classificationAnimaliaArhynchobdellidaOrobdellidae

http://zoobank.org/5A831984-50F6-433A-A058-ED2ECFF2DFDC

Figs 2 , 3 , 4 , 5

##### Diagnosis.

Body length of mature individual less than 5 cm. Somite IV uniannulate, somites VIII–XXV quadrannulate. Male gonopore in middle of XI b6, female gonopore in middle of XIII a1, behind gastropore, gonopores separated by 1/2 + 4 + 1/2 annuli. Clitellum in XI b5 to XIII a2. Pharynx reaching to XIII b5/b6–XIII/XIV. Gastropore conspicuous, in middle of XIII a1. Gastroporal duct bulbous, slightly winding at junction with gastropore. Paired epididymides in XIV b6–XV b5 to XVIII b6–XX a2/b5, occupying 16–20 annuli (four to five somites). Atrial cornua developed, ellipsoid or ovate.

##### Type materials.

**Holotype.** KUZ Z1652, dissected, collected from under a rock along a mountain trail at Mt. Kanmuriyama, Hatsukaichi, Hiroshima Pref., Japan (34.47325°, 132.10362°; Elev. 757 m; locality number 5), by TN on 25 April 2015. **Paratypes.** Two specimens from near the type locality, along a forest road, “Japan National Route 488”, Hatsukaichi, both dissected: KUZ Z1582, under a rock (34.50118°, 132.08933°; Elev. 790 m; locality number 5), by Yoshiko Yamane on 10 August 2014, and KUZ Z1654, under a rotten tree (34.50182°, 132.08961°; Elev. 791 m; locality number 5), by TN on 16 June 2015. For locality numbers, see Fig. 1.

**Additional materials.** In total four specimens were examined, all dissected. Three specimens collected from Hiroshima Pref., Japan: KUZ Z1360, from Hosomi, Kitahiroshima (34.685°, 132.292°; Elev. 470 m; locality number 4), by Yukiko Narahara on 9 July 2011; KUZ Z1380, from Mt. Azumayama, Hiwacho, Shobara (35.0639°, 133.0268°; Elev. 1010 m; locality number 2), by Ayane Maga on 3 October 2011; and KUZ Z1655, from under a rock along a mountain trail at Mt. Izaiyama, Hiwacho-Mitsugaichi, Shobara (35.00143°, 133.04640°; Elev. 906 m; locality number 3), by TN on 17 June 2015. KUZ Z1535, collected from under a rock along a mountain trail at Mt. Iimoriyama, Noigura, Kotoura, Tottori Pref., Japan (35.37603°, 133.59953°; Elev. 619 m; locality number 1), by TN on 11 December 2013. For locality numbers, see Fig. 1.

##### Etymology.

The specific name is a noun in the genitive case formed directly from the names of Ms Yukiko Narahara and Ms Ayane Maga, who collected specimens of this new species. Its stem is determined as “naraharaetmag” herein.

##### Description of holotype.

**Figure 2.**
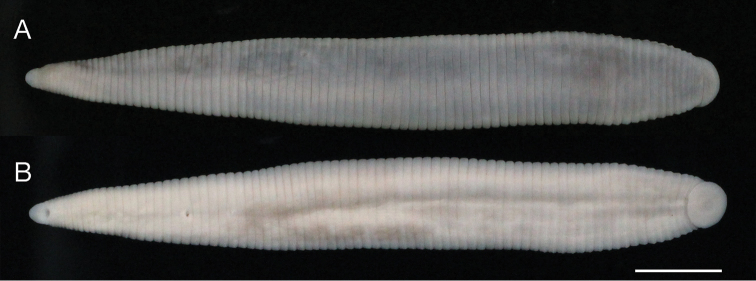
*Orobdella naraharaetmagarum* sp. n., holotype, KUZ Z1652. **A** Dorsal and **B** ventral views. Scale bar: 5 mm.

**Figure 3.**
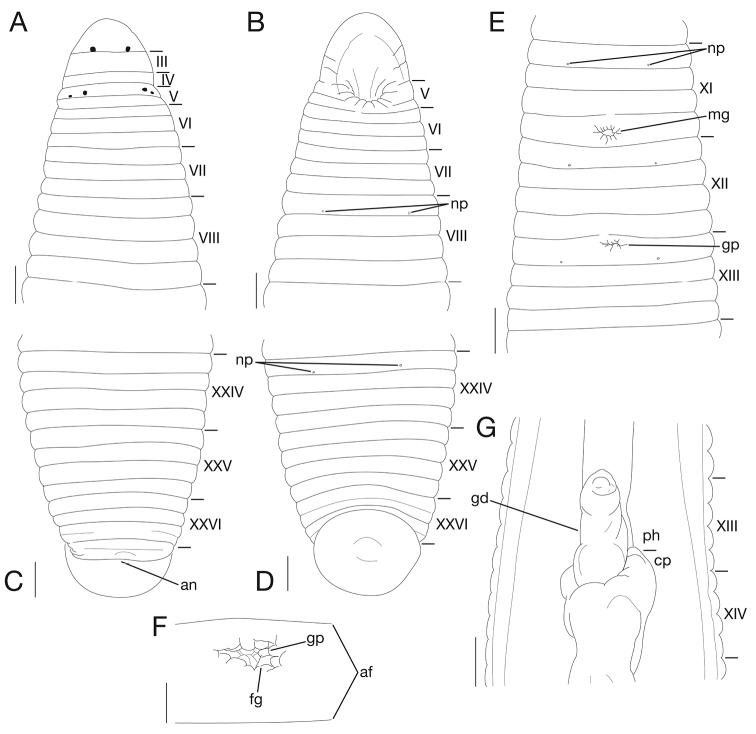
*Orobdella naraharaetmagarum* sp. n., holotype, KUZ Z1652. **A** Dorsal and **B** ventral views of somites I–VIII. **C** Dorsal and **D** ventral views of somites XXIV–XXVII and caudal sucker **E** Ventral view of somites XI–XIII **F** Ventral view of gastropore and female gonopore **G** Ventral view of gastroporal duct. Scale bars: 1 mm (**C–E, G**), 0.5 mm (**A, B**) and 0.25 mm (**F**). Abbreviations: af, annular furrow; an, anus; cp, crop; fg, female gonopore; gd, gastroporal duct; gp, gastropore; mg, male gonopore; np, nephridiopore; and ph, pharynx.

Body firm and muscular, elongate, with constant width in caudal direction, dorsoventrally compressed, BL 40.0 mm, BW 5.3 mm (Fig. 2). Caudal sucker ventral, elliptic, CL 2.4 mm, CW 3.0 mm (Figs 2B, 3D).

Somite I completely merged with prostomium (Fig. 3A). Somites II–IV uniannulate, II not separate from I (Fig. 3A). Somite V biannulate, (a1 + a2) > a3; a3 forming posterior margin of oral sucker (Fig. 3A, B). Somites VI and VII triannulate, a1 = a2 = a3 (Fig. 3A, B). Somites VIII–XXV quadrannulate, a1 = a2 = b5 = b6 (Fig. 3A–E). Somite XXVI dorsally triannulate, a1 > a2 < a3, a3 with slight furrow; ventrally biannulate, (a1 + a2) > a3, (a1 + a2) with slight furrow; (a1 + a2) being ventrally last complete annulus (Fig. 3C, D). Somite XXVII uniannulate with slight dorsal furrow; anus behind it with no post-anal annulus (Fig. 3C).

Male gonopore in middle of XI b6 (Fig. 3E). Female gonopore slightly anterior to middle of XIII a1, inconspicuous, located posterior to gastropore (Fig. 3E, F). Gonopores separated by 1/2 + 4 + 1/2 annuli (Fig. 3E).

**Figure 4.**
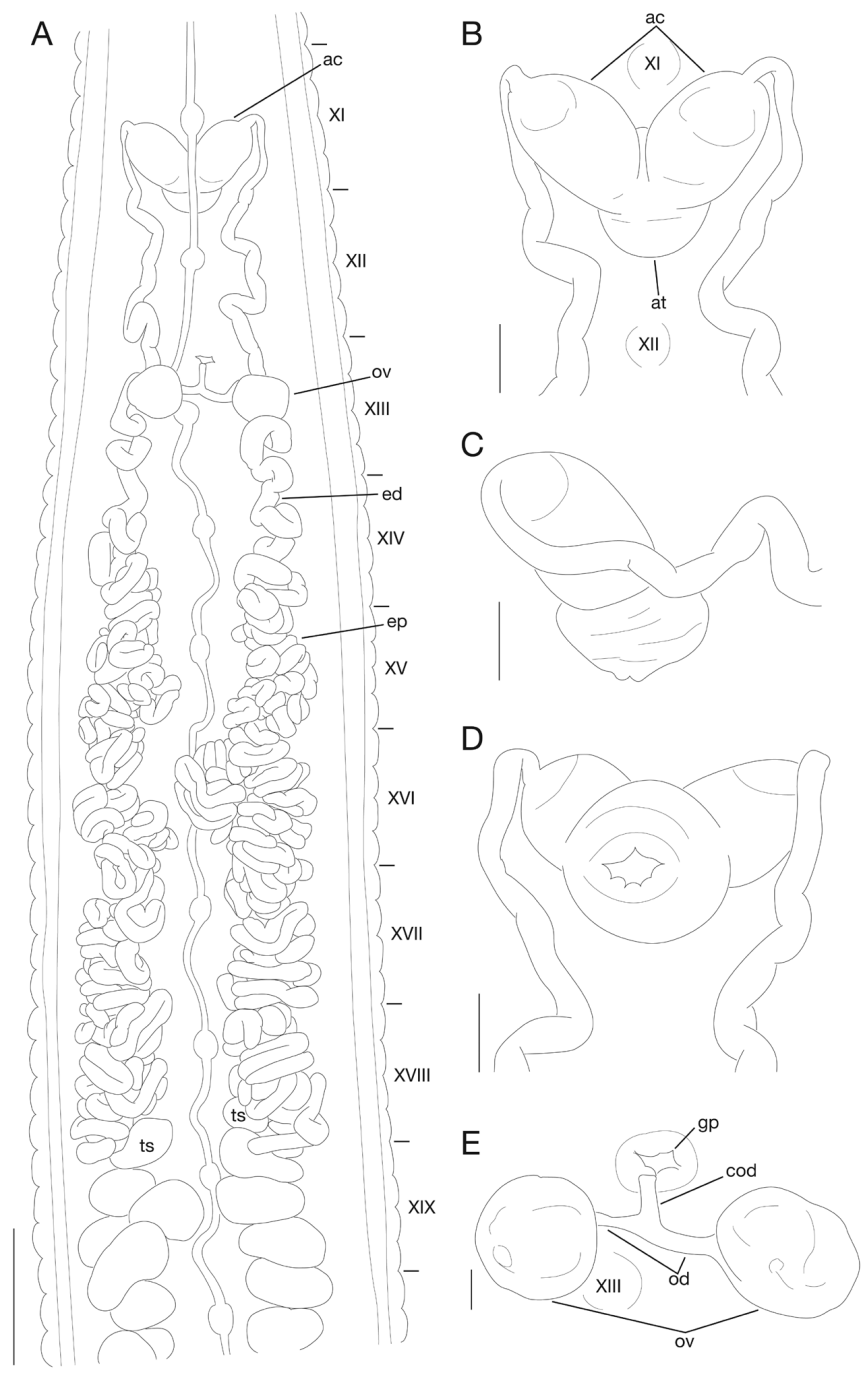
*Orobdella naraharaetmagarum* sp. n., holotype, KUZ Z1652. **A** Dorsal view of reproductive system including ventral nervous system. **B** Dorsal (including positions of ganglia XI and XII), **C** lateral, and **D** ventral views of male atrium. **E** Dorsal view of female reproductive system including position of ganglion XIII. Scale bars: 2 mm (**A**), 0.5 mm (**B–D**) and 0.25 mm (**E**). Abbreviations: ac, atrial cornua; at, atrium; cod, common oviduct; ed, ejaculatory duct; ep, epididymis; gp, gastropore; od, oviduct; ov, ovisac; and ts, testisac.

Anterior ganglionic mass in VI a2 and a3. Ganglion VII in a2. Ganglia VIII–X, of each somite, in a2. Ganglia XI and XII, of each somite, in a2 (Fig. 4A). Ganglion XIII in a2 and b5 (Fig. 4A). Ganglion XIV in a2 (Fig. 4A). Ganglia XV–XX, of each somite, in a1 and a2 (Fig. 4A). Ganglia XXI–XXIII, of each somite, in a2. Ganglion XXIV in a1. Ganglion XXV in b6 of XXIV and a1 of XXV. Ganglion XXVI in b5 and b6 of XXV. Posterior ganglionic mass in (a1 + a2) of XXVI.

Eyes in three pairs, first pair dorsally on posterior margin of II, second and third pairs dorsolaterally on posterior margin of V (a1 + a2) (Fig. 3A). Papillae numerous, minute, hardly visible, one row on every annulus.

Nephridiopores in 17 pairs, one each situated ventrally at posterior margin of a1 of each somite in VIII–XXIV (Fig. 3B, D, E).

Pharynx agnathous, euthylaematous, reaching to XIII b5/b6 (Fig. 3G). Crop tubular, acecate, reaching to XIX b5/b6. Gastropore conspicuous, ventral, slightly anterior to middle of XIII a1 (Fig. 3E, F). Gastroporal duct bulbous, slightly winding at junction with gastropore, joining with crop in XIV a1 (Fig. 3G). Intestine tubular, acecate, reaching to XXIV a1. Rectum tubular, thin-walled, straight.

Testisacs multiple (Fig. 4A); on right side, in XVIII b6 to XXIV a2, in total app. 28 testisacs, 1 in XVIII, 3 in XIX, 5 in XX, 4 in XXI, 6 in XXII, 7 in XXIII, 2 in XXIV; on left side, in XIX a1 to XXV a2, in total app. 27 testisacs, 5 in XIX, 6 in XX, 4 in XXI, 4 in XXII, 4 in XXIII, 3 in XXIV, 1 in XXV. Paired epididymides; right epididymis in XIV b6 to XVIII b6, occupying 17 annuli; left epididymis in XIV/XV to XVIII b6, occupying 16 annuli (Fig. 4A). Ejaculatory bulbs absent. Paired ejaculatory ducts; right duct in XI a2/b5 to XIV b6; left duct in XI a2/b5 to XIV/XV; coiled in position posterior to ovisacs; each duct crossing ventrally beneath each ovisac, then curved in position anterior to ovisacs; each widening from respective junction with epididymis, narrowing at junction with atrial cornua, then turning sharply inward toward atrial cornua without pre-atrial loop (Fig. 4A). Pair of muscular atrial cornua ellipsoid, in XI b5 and b6 (Fig. 4A–D). Atrium short, muscular, globular in XI b5 and b6 (Fig. 4B–D). Penis sheath and penis absent.

Paired ovisacs globular; right ovisac in XIII a2 and b5; left ovisac in XIII a1 and a2 (Fig. 4A, E). Oviducts, thin-walled, left oviduct crossing ventrally beneath nerve cord; both oviducts converging into common oviduct in XIII a2 (Fig. 4A, E). Common oviduct thin-walled, short, directly descending to female gonopore (Fig. 4E).

##### Variations.

BL 48.2 (KUZ Z1654)–33.0 (KUZ Z1360) mm, BW 4.4 (KUZ Z1535)–2.7 (KUZ Z1380) mm, CL 2.5 (KUZ Z1654)–1.2 (KUZ Z1380) mm, CW 3.0 (KUZ Z1654)–1.8 (KUZ Z1360, Z1380) mm. Somite XXVI triannulate, a1 = a2 < a3 (KUZ Z1380, Z1582); KUZ Z1360, Z1535, Z1654, Z1655, a3 with slight dorsal furrow. Somite XXVII uniannulate or biannulate (KUZ Z1360). Male gonopore generally in middle of XI b6, rarely slightly anterior or posterior to middle of XI b6. Female gonopore in middle of XIII a1, slightly anterior or posterior to middle of XIII a1. X b5 and XIII a2, respectively, being first and last annuli of clitellum. Eyes generally three pairs; KUZ Z1654, Z1655, first pair dorsally on anterior margin of III; KUZ Z1582, multiple eyes detected, one eye on left dorsal of II/III, one small eyespot on right dorsal of III, one small eyespot on left dorsal of III/IV, one small eyespot on right dorsal of IV, and two pairs of eyes dorsolaterally on posterior margin of V (a1 + a2). Pharynx reaching to XIII b5/b6–XIII/XIV. Crop reaching to XIX b5/b6–XX a1. Gastropore in middle of XIII a1, slightly anterior or posterior to middle of XIII a1. Gastroporal duct generally bulbous; KUZ Z1360, Z1582, tubular. Intestine reaching to XXIII/XXIV–XXIV/XXV. Testisacs multiple; right side app. 11–24 sacs in XIX b6–XX b5 to XXIV b5–b6; left side app. 11–23 sacs in XIX b6–XX a2 to XXIII b6–XXV a1. Paired epididymides; right epididymis in XV a1–XV b5 to XIX a2–XX a2/b5, occupying 17–20 annuli; left epididymis in XV a1–XV b5 to XIX b5–XX a2, occupying 17–20 annuli. Paired ejaculatory ducts, curved, loosely curved, or straight in position posterior to ovisacs. Atrial cornua ovate, fusiform, or ellipsoid in XI b5 and b6; KUZ Z1535 in XI b5–XII a. Atrium generally in XI b5 and b6; KUZ Z1535, Z1582 in XI b6. Paired ovisacs generally in XIII a2 and b5; KUZ Z1535, undeveloped, in XIII a2. Right or left oviduct crossing ventrally beneath nerve cord; KUZ Z1380 both oviducts converging into common oviduct in XIII a1/a2.

##### Coloration.

**Figure 5.**
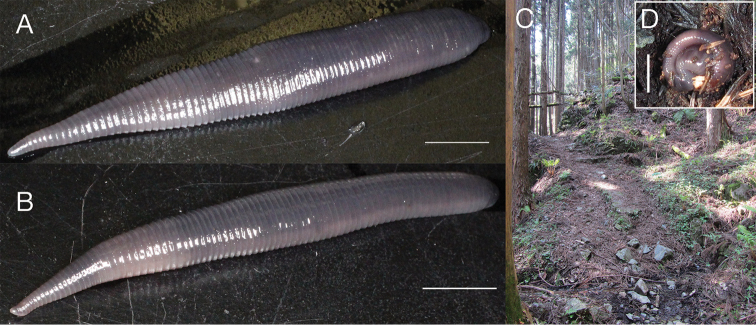
*Orobdella naraharaetmagarum* sp. n., holotype, KUZ Z1652 (**A**, **D**) and paratype, KUZ Z1654 (**B**). Habitat of holotype (**C**). **A** and **B** Dorsal views of live animals. **D** Live animal found curled up under a stone at the type locality. Scale bars: 5 mm.

In life, dorsal surface bluish gray (Fig. 5A, B), or gray; ventral surface reddish white or ash gray; clitellum, when obvious, whitish gray (Fig. 5B). Color faded in preservative; KUZ Z1535 with one dorsal black line from VIII b5 to XXVI a2.

##### Distribution.

This species was primarily collected in Hiroshima Prefecture in Chugoku District, Honshu, Japan, and in Tottori Prefecture. The lowest elevation among the localities was 470 m, and the highest was 1010 m. The locality data for this species suggested that it is distributed in mountainous regions in Chugoku District, Honshu, Japan.

##### Natural history.

This species was generally found curled up under rocks or rotten trees in moist mountainous habitats (Fig. 5 C, D). Oligochaete worms were sometimes observed in the digestive tract during specimen dissection. Therefore, this species is an earthworm-eater, as are the other known *Orobdella* leeches.

A mature leech with an obvious clitellum was collected on 16 June. Moreover, the holotype, which appeared to have a clitellum (Fig. 5A), was collected on 25 April. These findings suggest that the reproductive season of the new species begins in May and then continues at least to mid-to-late June.

##### Remarks.

The new species unambiguously belongs to *Orobdella* as it has all the generic diagnostic characteristics defined in Nakano et al. (2012): post-anal annulus absent; pharynx agnathous, euthylaematous; gastropore in XIII; gastroporal duct lying on female organ; gonopores separated by more than one full somite; testisacs multiple; male atrium in XI without penis sheath and penis; ovisacs globular in XIII; female median reproductive system essentially lacking.

The specimens were small (up to 48 mm). However, one leech (KUZ Z1654) was determined to be mature because it possessed an obvious clitellum. The holotype, which had a body length of 40 mm, also possessed a slightly developed clitellum and developed testisacs. Two specimens (KUZ Z1360, Z1582) possessed a tubular gastroporal duct. This tubular gastroporal duct was thought to be observed in immature individuals because these two specimens had undeveloped, undetectable testisacs.

Taxonomic studies (Nakano 2010, 2012b, 2014, Nakano and Gongalsky 2014, Nakano and Lai 2012, Nakano and Seo 2014) indicate that the new species differs from the seven other quadrannulate species (i.e., *Orobdella esulcata*, *Orobdella kawakatsuorum* Richardson, 1975, *Orobdella ketagalan* Nakano & Lai, 2012, *Orobdella koikei*, *Orobdella masaakikuroiwai*, *Orobdella tsushimensis* Nakano, 2011a, and *Orobdella whitmani* Oka, 1895) by the following combination of characteristics (Table 2): body length less than 5 cm, IV uniannulate, gonopores separated by 1/2 + 4 + 1/2 annuli, XXV quadrannulate, gastroporal duct bulbous, epididymides in XIV to XX, atrial cornua developed, ellipsoid or ovate. Among the above seven quadrannulate species, two species, *Orobdella koikei* and *Orobdella masaakikuroiwai*, are known to have body lengths shorter than 5 cm. *Orobdella naraharaetmagarum* can be distinguished from these two species by the annulation of XXV and the length of the epididymides.

The new species is distinguishable from the four sexannulate species *Orobdella dolichopharynx* Nakano, 2011b, *Orobdella ijimai* Oka, 1895, *Orobdella mononoke* Nakano, 2012a, and *Orobdella shimadae* Nakano, 2011b and the octannulate species *Orobdella octonaria* Oka, 1895, since *Orobdella naraharaetmagarum* possesses mid-body somites that are quadrannulate.

**Table 2.** Comparisons of morphological characters between *Orobdella naraharaetmagarum* sp. n. and seven quadrannulate congeneric species.

Character	*Orobdella naraharaetmagarum* sp. n.	*Orobdella esulcata* Nakano, 2010	*Orobdella kawakatsuorum* Richardson, 1975	*Orobdella ketagalan* Nakano & Lai, 2012	*Orobdella koikei* Nakano, 2012b	*Orobdella masaakikuroiwai* Nakano, 2014	*Orobdella tsushimensis* Nakano, 2011a	*Orobdella whitmani* Oka, 1895
**Body length of mature leech**	less than 5 cm	up to approx. 10 cm	up to approx. 10 cm	up to approx. 10 cm	less than 4 cm	less than 4 cm	up to approx. 10 cm	up to approx. 10 cm
**Annulation of IV**	uniannulate	uniannulate	biannulate	uniannulate	uniannulate	uniannulate	uniannulate	uni- or biannulate
**Number of annuli between gonopores**	1/2 + 4 + 1/2	2/3 + 4 + 1/3	6	1/2 + 4 + 1/2	1/2 + 4 + 1/2	1/2 + 4 + 1/2	1/2 + 5	1/2 + 4 + 1/2
**Annulation of XXV**	quadrannulate	quadrannulate	quadrannulate	quadrannulate	triannulate	quadrannulate	quadrannulate	quadrannulate
**Gastroporal duct**	bulbous	tubular, but bulbous at junction with gastropore	simple tubular	simple tubular	bulbous	bulbous	bulbous	bulbous
**Epididymides**	XV (posterior of XIV) to XX	XVI to XX	XVI to XVII	absent	XV to XX	XVI to XVIII	XVII to XIX	XVI to XVIII
**Atrial cornua**	developed, ellipsoid or ovate	developed, ovate	undeveloped	undeveloped	developed, ovate	developed, ovate	developed, ovate	developed, ovate

### Molecular phylogenies and genetic distances

**Figure 6.**
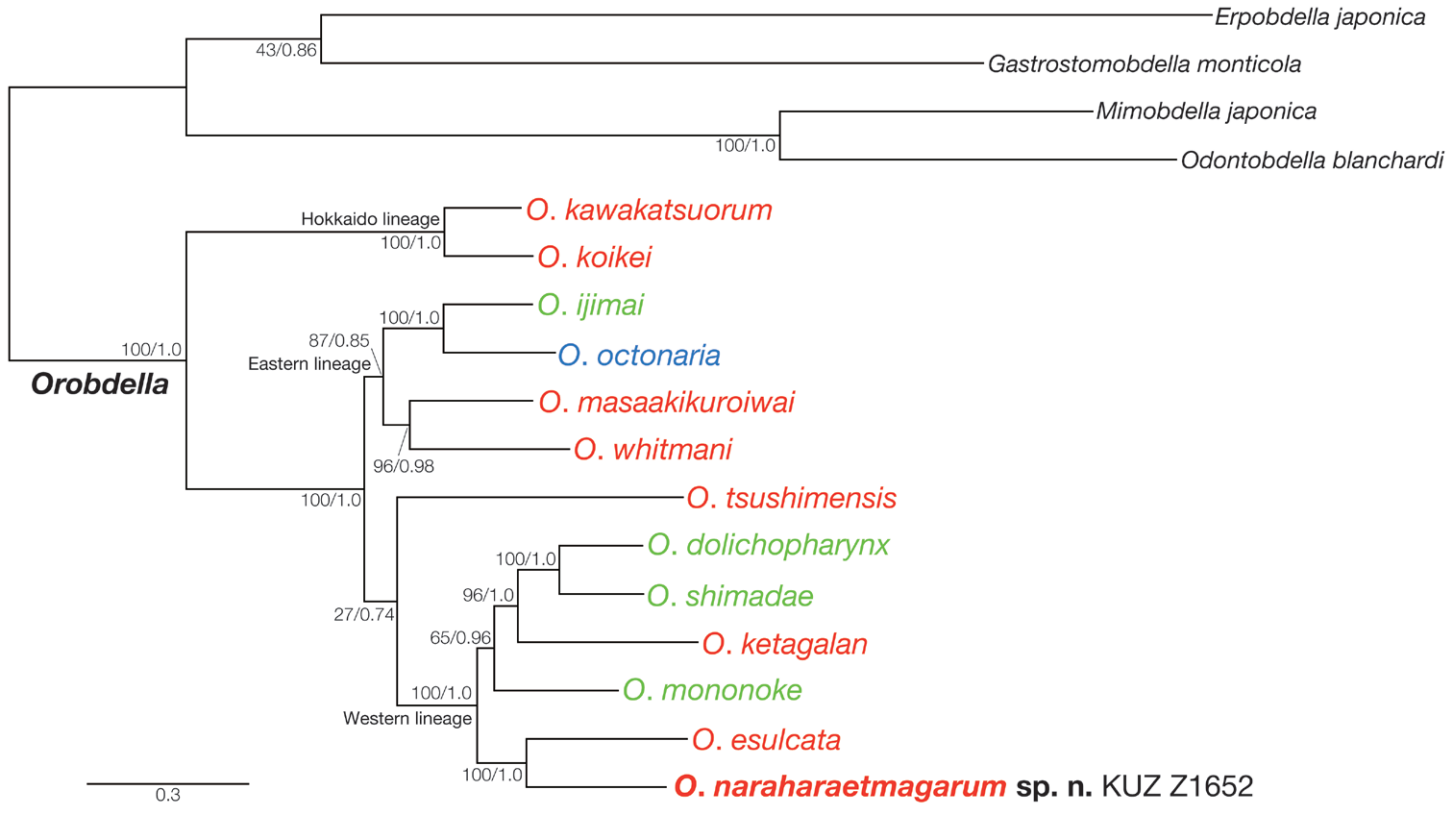
Bayesian inference tree for 5,192 bp of nuclear 18S rRNA and histone H3 and mitochondrial COI, tRNA^Cys^, tRNA^Met^, 12S rRNA, tRNA^Val^, 16S rRNA, tRNA^Leu^ and ND1 markers. Numbers on nodes represent bootstrap values for maximum likelihood and Bayesian posterior probabilities. A species name of *Orobdella* in red indicates a quadrannulate species; in green, sexannulate; and in blue, octannulate.

The BI tree (Fig. 6) for estimating the phylogenetic position of the new species had an identical topology to that of the ML tree (ln *L* = −24617.61; not shown). The monophyly of *Orobdella naraharaetmagarum* and *Orobdella esulcata* was strongly supported (BS = 100%, BPP = 1.0).

**Figure 7.**
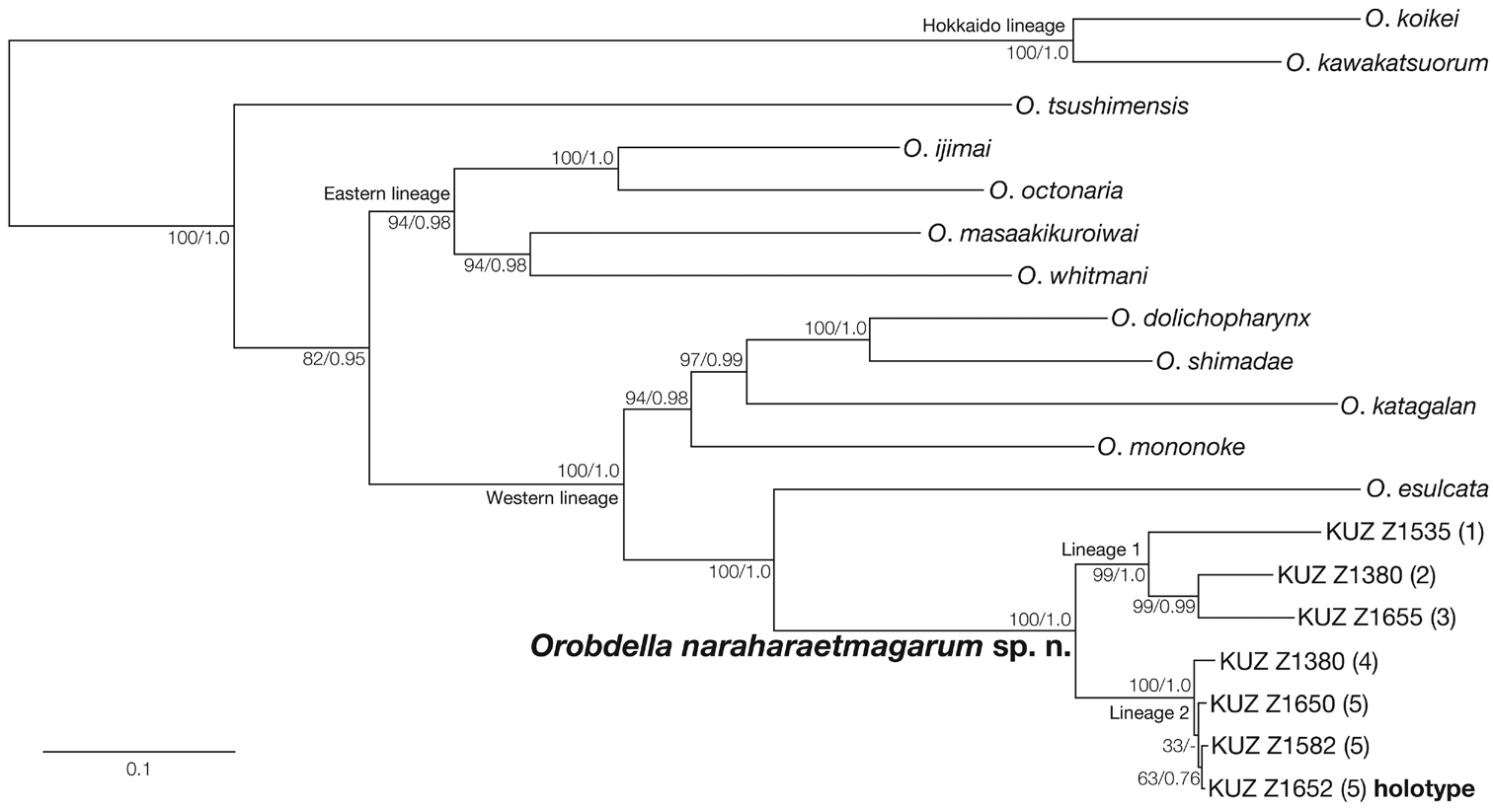
Maximum likelihood tree (ln *L* = −15057.02) for 3,008 bp of mitochondrial COI, tRNA^Cys^, tRNA^Met^, 12S rRNA, tRNA^Val^, 16S rRNA, tRNA^Leu^ and ND1 markers. Numbers on nodes represent bootstrap values for maximum likelihood and Bayesian posterior probabilities.

The ML tree (ln *L* = −15057.02) (Fig. 7) used to construct the phylogenetic relationships of the new species had an almost identical topology to that of the BI tree (not shown). The monophyly of the specimens identified as *Orobdella naraharaetmagarum* was recovered (BS = 100%, BPP = 1.0). This clade was divided into two subclades (hereafter lineages 1 and 2). The monophyly of lineage 1 was strongly supported (BS = 99%, BPP = 1.0). Lineage 1 comprised three specimens: KUZ Z1380 (locality number 2 in Fig. 1), Z1535 (locality number 1), and Z1655 (locality number 3). KUZ Z1380 and Z1655 formed a monophyletic group (BS = 99%, BPP = 0.99). The monophyletic lineage 2 (BS = 100, BPP = 1.0) contained four individuals: KUZ Z1380 (locality number 4), Z1582, Z1650, and Z1652 (locality number 5). In the ML phylogeny, the three specimens KUZ Z1582, Z1650, and Z1652 formed a monophyletic lineage, although this relationship was not supported (BS = 33%). The monophyly of KUZ Z1582 and Z1652 was also not fully supported (BS = 63%, BPP = 0.76).

The pairwise COI uncorrected *p*-distance within *Orobdella naraharaetmagarum* was 0.6–4.7% (mean = 3.3%) (Table 3). The genetic distance between lineages 1 and 2 was 4.1–4.7% (mean = 4.4%). The genetic divergences within lineages 1 and 2 were 3.1–4.2% (mean = 3.8%) and 0.6–1.4% (mean = 1.1 %), respectively. In addition, that between *Orobdella naraharaetmagarum* and *Orobdella esulcata* was 8.4–9.1% (mean = 8.9%)

**Table 3.** Uncorrected *p*-distances for the 1266 bp for the COI sequences of *Orobdella naraharaetmagarum* sp. n. specimens, with associated collection locality numbers (see Fig. 1).

Specimen (locality number)	1	2	3	4	5	6	7
1: KUZ Z1535 (1)							
2: KUZ Z1380 (2)	0.042						
3: KUZ Z1655 (3)	0.041	0.031					
4: KUZ Z1360 (4)	0.045	0.043	0.041				
5: KUZ Z1654 (5)	0.042	0.042	0.042	0.011			
6: KUZ Z1582 (5)	0.047	0.043	0.042	0.014	0.009		
7: KUZ Z1652 (5)	0.047	0.046	0.044	0.013	0.010	0.006	

## Discussion

The obtained molecular phylogenies showed that the present specimens formed a well-supported clade. In addition, the mean value of the COI uncorrected *p*-distance among the individuals was 4.4%. This value indicated a clear gap between the present specimens and the closest congener, *Orobdella esulcata*. Therefore, all of the specimens examined can be considered to belong to the same species, *Orobdella naraharaetmagarum*.

Although the precise phylogenetic position of *Orobdella tsushimensis* from the Korean Peninsula and the adjacent islets could not be determined in the obtained phylogenies (see Fig. 6), they showed that the genus *Orobdella* comprises three clades: a Hokkaido lineage including *Orobdella kawakatsuorum* and *Orobdella koikei*; an eastern lineage consisting of four species, *Orobdella ijimai*, *Orobdella masaakikuroiwai*, *Orobdella octonaria*, and *Orobdella whitmani*, distributed in eastern Honshu; and a western lineage containing five previously described species, *Orobdella mononoke*, *Orobdella dolichopharynx* and *Orobdella shimadae* from the Nansei Islands, *Orobdella ketagalan* from Taiwan, and *Orobdella esulcata* recorded in Kyushu, Japan. The present phylogenies demonstrated that *Orobdella naraharaetmagarum* was a member of the last clade, and formed a monophyly with *Orobdella esulcata* with strong support. Therefore, the range of the western lineage group covers the area from Chugoku District, at the western tip of Honshu, to Taiwan.

As indicated in Figure 7, *Orobdella naraharaetmagarum* was divided into eastern (lineage 1; locality numbers 1–3) and western (lineage 2; locality numbers 4, 5) phylogroups. The COI uncorrected *p*-distances within lineage 1 were higher than those within lineage 2. The calculated genetic divergences between the three specimens collected from Mt. Kanmuriyama (locality number 5; KUZ Z1582, Z1652, and Z1654) and one individual, KUZ Z1360, from Kitahiroshima (locality number 4) was 1.1–1.4%. The geographic distance between these two collection localities is ca. 28 km. In comparison, the genetic distance between KUZ Z1380 collected on Mt. Azumayama (locality number 2) and KUZ Z1655 from Mt. Izaiyama (locality number 3) was greater than this value (3.1%), although these two localities are separated only by ca. 7 km. These phylogenetic relationships and genetic divergences implied that leeches belonging to lineage 2 dispersed more recently and rapidly than those of lineage 1. Such discordance between the COI genetic divergences and geographical distances was also seen in the small species *Orobdella masaakikuroiwai* (Nakano 2014). Further molecular phylogenetic studies will help to reveal the biogeographical history of the *Orobdella* leeches.

The phylogenetic position of *Orobdella naraharaetmagarum* also indicated that the small size of the mature leeches evolved in parallel within *Orobdella*, as mentioned in Nakano (2014). According to the obtained molecular phylogenies and the phylogenetic trees from studies (Nakano 2012b, 2014), each of the three small species, *Orobdella koikei*, *Orobdella masaakikuroiwai*, and *Orobdella naraharaetmagarum* may have diverged from a single large quadrannulate species. As with the other two small species (Nakano 2012b, 2014), *Orobdella naraharaetmagarum* is also distributed sympatrically with undescribed large quadrannulate species in Chugoku District (Nakano, unpublished data). Therefore, further systematic studies should be carried out to reveal the species diversity and evolutionary history of the genus *Orobdella*.

## Supplementary Material

XML Treatment for Orobdella naraharaetmagarum
